# The Alleviation of Metal Stress Nuisance for Plants—A Review of Promising Solutions in the Face of Environmental Challenges

**DOI:** 10.3390/plants11192544

**Published:** 2022-09-28

**Authors:** Mateusz Labudda, Kinga Dziurka, Justyna Fidler, Marta Gietler, Anna Rybarczyk-Płońska, Małgorzata Nykiel, Beata Prabucka, Iwona Morkunas, Ewa Muszyńska

**Affiliations:** 1 Department of Biochemistry and Microbiology, Institute of Biology, Warsaw University of Life Sciences-SGGW, Nowoursynowska 159, 02-776 Warsaw, Poland; 2 Department of Biotechnology, The Franciszek Górski Institute of Plant Physiology, Polish Academy of Sciences, Niezapominajek 21, 30-239, Kraków, Poland; 3 Department of Plant Physiology, Poznań University of Life Sciences, Wołyńska 35, 60-637 Poznań, Poland; 4 Department of Botany, Institute of Biology, Warsaw University of Life Sciences-SGGW, Nowoursynowska 159, 02-776 Warsaw, Poland

**Keywords:** abiotic stress, adaptation, priming, defense mechanisms, metallophyte, oxidative stress, phytoremediation, tolerance

## Abstract

Environmental changes are inevitable with time, but their intensification and diversification, occurring in the last several decades due to the combination of both natural and human-made causes, are really a matter of great apprehension. As a consequence, plants are exposed to a variety of abiotic stressors that contribute to their morpho-physiological, biochemical, and molecular alterations, which affects plant growth and development as well as the quality and productivity of crops. Thus, novel strategies are still being developed to meet the challenges of the modern world related to climate changes and natural ecosystem degradation. Innovative methods that have recently received special attention include eco-friendly, easily available, inexpensive, and, very often, plant-based methods. However, such approaches require better cognition and understanding of plant adaptations and acclimation mechanisms in response to adverse conditions. In this succinct review, we have highlighted defense mechanisms against external stimuli (mainly exposure to elevated levels of metal elements) which can be activated through permanent microevolutionary changes in metal-tolerant species or through exogenously applied priming agents that may ensure plant acclimation and thereby elevated stress resistance.

## 1. Introduction

**Figure 1:**
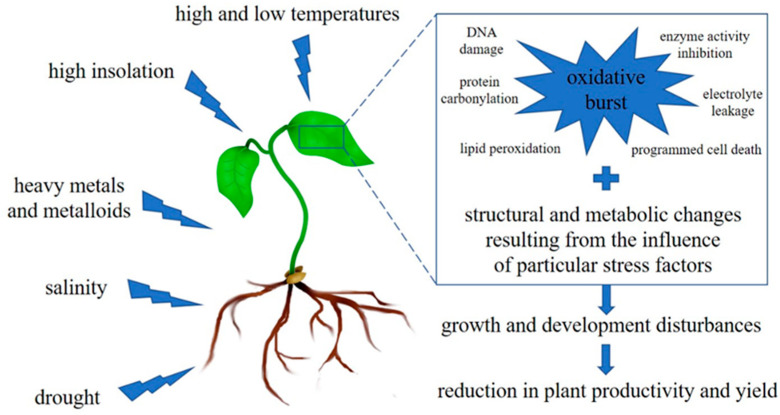
A simplified diagram of complex plant responses to stressful environmental stimuli: oxidative burst and its consequences as a universal reaction to different stressors are shown, as well as stressor-dependent reactions leading to plant growth retardation and a decline in productivity.

Rapid industrialization and urbanization, chemicalization of agriculture, and the lack of a proper attitude to the surroundings in which we live are the main causes of unpredictable climate changes, as well as the deterioration of natural environments and ecosystems [1]. As a consequence of such imprudent human domination of the Earth, plants are constantly exposed to a wide array of adverse environmental events, including water deficits, salinity, imbalances in elements (resulting from their deficiency and/or pollution), extremes of temperature, ultraviolet radiation, etc. All the above-mentioned physical and chemical factors, collectively referred to as abiotic stress, may occur singly, sequentially, or simultaneously, and their effects may also act synergistically or additively on plant fitness [2]. Moreover, the effect of each stress factor depends on its intensity and the exposure time of the plants. Despite the impact of such a wide variety of stressors, plant exposure to any of them has one similar outcome, namely the overgeneration of reactive oxygen species (ROS) that are responsible for oxidative damage of cellular components such as proteins, lipids, nucleic acids, carbohydrates, and other metabolites [3,4,5]. Therefore, as shown in Figure 1, oxidative stress is a secondary but common reaction of plants subjected to various factors which, by interacting with each other, contribute to disturbances in the optimal growth and development of plants, finally leading to a considerable reduction in their productivity and yield [6,7].

It should be emphasized that, among the various xenobiotics released to the environment due to anthropogenic activity, heavy metals and metalloids, classified also as metallic trace elements because of their presence at trace concentrations (parts per billion or less than 10 parts per million) in various matrices, have pulled ahead of other well-known contaminants such as plant protection products and carbon and sulfur dioxides [8]. Environmental pollution with metals is particularly prominent in point source terrains such as metalliferous mines, smelters and foundries, and other metal-based industrial operations. However, among the sources of these most common inorganic contaminants, fossil fuel burning and the use of fertilizers, pesticides, livestock manure, municipal wastes, and sewage should also be mentioned [9]. The problem of heavy metal accumulation may be aggravated by salinity stress, which causes disturbances in the homeostasis of macro- and micro-elements in the soil and facilitates metallic ion uptake by plants [10,11]. Such changes in soil composition are peculiarly important in the case of crops for which a sufficient supply of essential elements has to be ensured, whilst potentially toxic elements should be present only at very low levels. Since heavy metals are now ranked in second place when taking into account the degree of risk they pose to the human population all over the world, in recent years there has been increasing concern about environmental contamination from them [12].

Metallic trace elements can spread over long distances through interactions with wind, surface and ground waters, and herbivores. These metals pose a serious threat to our health due to food chain accumulation, dust inhalation, and skin contact which result in cardiovascular, respiratory, and neurodegenerative diseases [13]. They also have a negative impact on the majority of plant species and other living organisms. In the concentrations exceeding the maximum tolerable amount, they cause disturbances in the ultrastructure of cells and affect physiological and biochemical processes such as the biosynthesis of chlorophylls, photosynthetic capacity, transpiration, nutrient and water uptake, and the activity of enzymes involved in various metabolic pathways, as well as lead to increased ROS formation by direct involvement in redox reactions (in the case of highly reactive metals) or indirectly through depletion of antioxidant pools [5,9,14,15,16,17]. All these cellular effects result in morphological changes, such as shortening of shoots and roots, necrotic and chlorotic stains, decreases in leaf number and size, and premature aging [5,18,19]. As a consequence, it leads to limiting the productivity of agricultural crops [20]. The danger of metallic elements lies also in the fact that many of them are dispersed in the environment for a long time and, therefore, they are considered to be persistent [12]. As an example, half-life time varies from 75 to 380 years for cadmium (Cd) and from 1000 to 3000 years for copper (Cu), nickel (Ni), lead (Pb), zinc (Zn), and selenium (Se) in the soils of temperate climates [21].

Recently, research on the mechanisms by which plants recognize and cope with toxic metals and other stressful and dynamic circumstances has undergone a very exciting period leading to significant breakthroughs. The development of knowledge in this field is necessary to relieve the pressure of environmental changes and to ensure global food security for an increasing population, as well as to restore areas degraded by human activity. Since it is well known that plants have developed different adaptation strategies which can occur as a result of adaptation and acclimation, the purpose of this concise review is to indicate what can make the life of stressed plants a little easier, especially in respect to metallic trace elements. We have highlighted only two main possibilities, although many more issues are taken into consideration in the current research. The first one is based on natural defense mechanisms arising through evolutionary changes, the understanding of which enables the development of new strategies to alleviate metal danger for both plants and surroundings (and/or the improvement of those remediation techniques that already exist). The second one refers to the use of novel priming techniques that may provide plants with intracellular acclimation and thereby enhanced stress tolerance. Both of them represent the latest solutions for sustainable, cost-effective, and efficient approaches to environmental challenges.

## 2. Functional Traits of Plants Developed in Response to Severe External Pressures

Climate change has caused serious impacts on the ecosystem, including devastating its stability and affecting biodiversity. Plants, as an important component of terrestrial ecosystems, respond to climate change in an all-round way; therefore, changes in the functional traits of plants can be indicative of climate changes. The novel developmental direction of this research is to determine the interrelationships among various indicators based on physiological, biochemical, and ecological plant characteristics and to establish a network indicator system from individual plants and communities towards ecosystem functions.

Since plants are unable to avoid environmental stressors due to their sessile lifestyle, they have evolved effective mechanisms to combat stress which ensure their survival in uncomfortable conditions. Defense response can be attributed to phenotypic plasticity leading to changes within a single organism, that are reversible and result from subsequently occurring, occasional stress events (‘priming’), or from chronic exposure to a new environment, to which plant metabolism adjusts (‘acclimation’) [22,23]. Both of these terms differ from ‘adaptation’, which describes permanent genotypic changes resulting in phenotypic traits that improve plant fitness or survival over multiple generations [24]. Morphological, anatomical, and physiological adaptations are characterized for metallophytes that have been gradually developed in habitats naturally or artificially enriched with metallic elements. Although in these first conditions metal tolerance may evolve over thousands or even millions of years, on human-influenced metalliferous soils it may be achieved in a relatively short time, i.e., less than 100–150 years [25]. Such genetically altered ecotypes of common species (i.e., pseudometallophytes or facultative metallophytes), as well as genera restricted only to metalliferous soils (i.e., obligate or absolute metallophytes), exhibit a higher toxicity threshold or even slightly beneficial metal effects compared to their counterparts from unpolluted areas due to a special tolerance mechanism which is not available to non-metalliferous genotypes [26].

### 2.1. Specific Characteristics of Metal-Tolerant Species and Their Application in Soil Remediation

Metallophytes utilize several adaptation mechanisms to control the uptake, mobility, and activity of potentially toxic ions in the cell. Firstly, modifications to cell wall components and structure favor the retention of metals and provide a mechanical and chemical barrier against their free penetration into the protoplast [27,28]. Similarly, various membrane transporters belonging to the following families: HMAs (heavy metal ATPases, also known as P-type ATPase), NRAMP (natural resistance-associated macrophage protein), CDF (cation diffusion facilitators), YSL (yellow stripe-like), ABC (ATP-binding cassette), COPT (copper transporter), and ZIP (zinc-regulated transporter, iron-regulated transporter-like protein), play an important role in the regulation of toxic ion influx into the protoplast and organelles, and therefore have been extensively discussed in recent research and numerous review articles [7,29,30,31]. Subsequently, in the cytosol, harmful ions are effectively detoxified and stored in places safe for metabolism in order to prevent deleterious physiological damage. The important cytoplasmic ligands responsible for the chelation and neutralization of metallic elements include phytochelatins, glutathione (GSH), amino acids, and organic acids [14,26,32]. In turn, metal sequestration may take place in the vacuole, dictiosomal vesicles, or the endoplasmic reticulum [33,34]. From an organismic point of view, ions can be withdrawn into aging leaves and trichomes or drawn outside by secretary glands, which has been observed in *Arabidopsis thaliana* and *A. halleri* [35], *Alyssum montanum* [36], and *Biscutella laevigata* [37].

Other cellular features that make the life of metallophytes easier are related to efficient antioxidant defense systems that confront oxidative stress. Cell redox homeostasis is kept by a synchronous action of various enzymes, such as superoxide dismutase (SOD), catalase (CAT), peroxidases (POD; such as guaiacol peroxidase, GPOX, glutathione peroxidase, GPX, and ascorbate peroxidase, APX), glutathione *S*-transferase (GST), glutathione reductase (GR), and nonenzymatic antioxidants, such as ascorbate (AsA), glutathione (GSH), carotenoids (CAR), α-tocopherols, phenolics, and amino acids such as proline [4]. Although oxidative stress as a reaction to metals is one of the most studied issues recently [3,5,38,39], ROS transformation pathways, as a basis of adaptation to their excess amounts, have not been frequently compared between the representatives of different species sharing the same ecological niches or described for ecotypes of the same species representing different habitats. In this regard, our previous studies on serpentine and calamine ecotypes of *Silene vulgaris* (Caryophyllaceae) and the calamine ecotype of *Alyssum montanum* (Brassicaceae) have shown both species- and ecotype-dependent features [14,18,19,36,40,41]. The response of *S. vulgaris* to metallic elements was mainly related to the activity of antioxidant enzymes during in vitro cultivation on media enriched with Zn, Pb, and Cd at the same concentration as in the post-industrial habitat of the calamine ecotype [41]. In turn, the response of *A. montanum* was associated with the transformations of phenolic compounds, which, in the metallicolous ecotype, led to the synthesis of phenolic acids with a high ability to ROS scavenging and, in the non-metallicolous ecotype, to the synthesis of other compounds not involved in alleviating oxidative stress [19]. Interestingly, the common reaction of the metallicolous *S. vulgaris* and *A. montanum* individuals was the activity of GPX. Nevertheless, the importance of this enzyme in particular ecotypes differentiated both calamine specimens from the serpentine ones. In the former, the increased activity of this enzyme correlated with the increased accumulation of phenylpropanoids which, acting together, contribute to the formation of a lignified cell wall preventing the easy penetration of ions into the protoplast; whereas GPX activity in the serpentine ecotype provided only ROS neutralization [36,41]. Curiously, we have also found that the exposure of the calamine ecotype of *S. vulgaris* to the concentration of metallic elements reflecting their level in the zinc–lead substrate resulted in a significant increase in the efficiency of all analyzed components of the antioxidant apparatus. As a consequence, the studied ions stimulated the growth of calamine specimens, which was manifested in accelerated growth and biomass accretion [40,41]. It is therefore likely that the trace element ions, at the doses which the calamine ecotype has adapted to in the selection process, play a pivotal physiological role, perhaps even as micro- or ultra-elements.

The above-mentioned mechanisms guarantee a high propensity of metallophytes to take up metallic trace elements; however, tolerant species, ecotypes, or particular populations differ in their degree of accumulation and the element distributions in their organs, even if they grow on the same soil. Furthermore, the enhanced ability of metal-tolerant species to accumulate one metal does not mean that other ions will be stored with the same intensity and distributed over the organs in a similar way [32]. As proposed by Baker [42], plants appearing in metal-enriched environments can be divided, on the basis of the relationship between ion content in tissue and soil, into:

(1) ‘excluders’ that detoxify most of the toxic ions in roots and minimize their translocation to shoots; for these plants the accumulation coefficient, i.e., the ratio of metal concentration in the shoot to the soil, is always lower than one;
(2) ‘indicators’, whose shoots contain a similar concentration of metals as the soil (the accumulation coefficient is close to one);
(3) ‘accumulators’, which are characterized by effective metal uptake, transport, and storage in shoots (the accumulation coefficient is higher than one); among them, approximately 720 species are considered to be hyperaccumulators that are able to accumulate extraordinary amounts of metallic ions without suffering any phytotoxic effects [43].

The amazing biology and behavior of metallophytes, in respect to metal accumulation and detoxification, make them to useful in various phytoremediation techniques. It is plant-based, environmentally friendly, non-invasive, and low-cost technology which is applied to remediate contaminated soils by accumulation, immobilization, or degradation of these pollutants [44]. In phytoextraction, which constitutes the most popular method of phytoremediation relying on the total removal of contaminants from the environment, hyperaccumulating plants may work the best due to their ability (about 100–1000-fold higher than in other plants) regarding effective uptake and translocation of metallic elements [45]. Many studies have demonstrated the phytoextraction potential of metallophytes from various genera, such as *Alyssum* *murale* [46], *Arabidopsis halleri* [35], *Biscutella laevigata* [37], and *Stackhousia tryonii* among others [47]. Nowadays, phytoextraction achieves two goals at once. It is not only exploited to clean up soil, but also to mine metal (so-called phytomining), mainly in places where the use of conventional methods for ore exploitation is economically unprofitable. As an example, the cultivation of Ni-hyperaccumulators *Alyssum corsicum* and *A. murale* allows the extraction of about 400 kg of Ni per hectare [48]. In turn, metal excluders are excellent candidates for phytostabilization, which is aimed at reducing metal mobility in the soils in order to prevent them leaching deeper into the ground water and to also prevent the dust blowing into the atmosphere [44]. Despite metal stabilization, this technique involves the permanent establishment of a vegetative cover, which performs anti-erosive and soil-forming functions. Currently, some studies have indicated that the recovery of vegetation on heavy metal polluted terrains should be performed by native metalliferous species, which spontaneously occur on degraded areas and are thus better adapted to local ecological conditions than introduced ones [49]. Such an approach was first used in the 1960s, when Zn-Pb tolerant populations of *Agrostis tenuis, A. stolonifera, Anthoxanthum odoratum, Festuca rubra*, and *F. ovina* were investigated [50]. Recently, the potential of native metal-tolerant species for revegetation has been successfully verified for *Agropyron smithii* and *Artemisia tridentate* [51], *Lygeum spartum* [52], *Achillea wilhelmsii* [53], *and* *Matthiola dagestanica* and *Draba stylaris* [46]. The usefulness of metallophytes for revegetation and the phytostabilization of Zn-Pb rich soils in the Olkusz Ore-bearing Region, one of the biggest industrial areas in Poland, has been also proven in our earlier studies for *Biscutella laevigata* [49,54], *Dianthus carthusianorum* [49], *Gypsophila fastigiata* [55], and *Silene vulgaris* [56]. Undoubtedly, phytoremediation combined with the biological reclamation of destroyed or degraded ecosystems may constitute a new and safe opportunity for humans to positively interact with the environment.

### 2.2. Relationship between Chosen Metal Tolerance Traits and Other Stresses

Besides evolving metal tolerance, metallicolous species or their ecotypes were co-selected for tolerance to other adverse site conditions because soils contaminated with heavy metals are often salinized and dry [49]. Therefore, metallophytes share tolerance mechanisms with other specialized groups of plants, which makes their biology even more interesting.

Apart from activation of the antioxidant defense system constituting the basic response to various types of stress, metal-tolerant species exhibit specific adaptations that ensure a high degree of resistance to salinity and drought as well, both of which may cause a lack or deficiency of water for plants. The increased resistance to water deficit in metallophytes may result from their ability to accumulate toxic ions in large amounts. Since metallic ions can be preferentially accumulated within epidermal leaf cells, reduced cuticular transpiration can be achieved [47]. Furthermore, one hypothesis justifying reasons for metal (hyper)accumulation postulates that elements stored within cells overcome the effect of water constraints by acting as an osmolyte [45]. Confirmation of the osmoregulatory role of metals can be found in a study conducted by Bhatia et al. [47], who proved that Ni content in the shoots of *Stackhousia tryonii*, a Ni hyperaccumulator, increased significantly as the soil moisture levels decreased. Another excellent osmolyte that also accrues during metal stress is proline. This important amino acid contributes to maintaining water balance and cell turgor through osmotic regulation, and also contributes to the stability of cell membranes by preventing electrolyte leakage, which in turn can help during water deficit [57,58]. Besides osmoregulation function, proline also acts as a metal chelator and an antioxidative defense molecule which prevents oxidative burst due to ROS scavenging, thus mitigating a wide array of adverse effects from toxic ions [4,41,59]. Subsequent features of metallophytes which may provide simultaneous protection against water losses or their better adjustment to drought are related to morpho-anatomical structure. Leaves of metal-tolerant species often have reduced transpiration surfaces, are less numerous, narrower, thicker, and waxy, and possess a limited number of stomata and increased mesophyll cell size [17,60,61]. Furthermore, plants from metalliferous areas, probably in response to dry substrate, may produce deeper roots covered with dense root hairs; however, root architecture and size do not form a rule enabling metal-tolerant individuals to be distinguished from non-tolerant ones [32].

Metallophytes also show some similarities with halophytes, natives of saline soils mostly rich in sodium (Na^+^) and chloride (Cl^−^) ions. Both these specialized groups of plants possess specific and more common functional mechanisms of tolerance towards numerous stresses, which refer not only to strong antioxidant defense systems and the synthesis of compatible solutes, but also to ion sequestration and detoxification pathways [62]. The vacuolar compartmentalization of salt and heavy metals through the enhanced activity of membrane transporters is one of them [63]. Nevertheless, it has not been fully explained if the same proton pumps are involved in this process, although the role of vacuolar H^+^-ATPase was proven to protect against salt and Cd stress in the halophyte *Tamarix hispida* [64]. Moreover, halophytes, similarly to metal-tolerant species, are able to excrete excess deleterious ions from photosynthetically active tissues on leaf surfaces by different structures, such as salt glands, bladders, and trichomes; however, they are not specific to salt alone, as other toxic ions can be also removed in this way [62]. As an example, *Armeria maritima* ssp. *halleri*, an obligate metallophyte, can remove Cu ions via salt glands [65], while *Limoniastrum monopetalum*, a halophytic plant, uses these structures to excrete salt, Cd, and Pb as a detoxification mechanism [66]. On the other hand, all the above-mentioned mechanisms indicate clearly that the adaptation of halophytes for survival in the presence of high salt concentrations may also confer their tolerance to metallic elements. For this reason, halophytes can be good candidates for the phytoremediation of heavy metal polluted soils. These specimens with exclusion ability, rapid growth, and deep root systems can form dense vegetation cover and therefore be utilized for the purposes of phytostabilization. *Atriplex halimus* [67], *Cochearia anglica, C. x hollandica, C. danica, C. py-renaica* [63], and others are good examples (Table 1). Among halophytes, species that are able to accumulate both heavy metals and salt in extraordinary amounts in the shoots without suffering phytotoxic effects can be also found. One of the most effective in removing toxic ions seems to be an annual halophyte, *Chemopodium botrys*, which accumulates several times more Cd than *Noccaea caerulescens*, a well-known hyperaccumulator of Cd and Zn [68]. The study of Mazharia and Hoameed [68] indicated that the total amount of Cd removed by shoots of *Ch. botrys* was 120 g/ha; whereas the average Cd extraction ability of *N. caerulescens* may stay at a level of about 35 g/ha. Such salt/metal-accumulating species are extremely important for the decontamination of metal polluted saline soils, although recent findings also encourage their use for reclamation of purely saline soils, mainly in arid and semiarid regions [69]. Some more examples of halophytic species and their potential usefulness in particular soil phytoremediation methods are shown in Table 1, whilst the HALOPH database, which is available at http://www.sussex.ac.uk/affiliates/halophytes/ (accessed on 3 August 2022), presents probably the largest collection of halophyte examples for various applications. In turn, more aspects of halophyte responses to metallic elements (including common and specific mechanisms of metal and salt tolerance in this group of plants), their potential utilization for the phytoremediation of metal-contaminated soils, and their relevance to the phytodesalination of saline lands have been broadly discussed in some recently published reviews and books [62,70,71,72].

## 3. Chemical and Physical Agents for Enhancing Plant Resistance to Abiotic Stress

Until now, many different techniques developed by humans have been applied to improve plant tolerance to abiotic stress factors. Some of them are based on conventional breeding; however, they have many limitations, such as being time-consuming, possessing the possibility of transferring numerous undesirable genes along with desirable ones, and having no guarantee of obtaining a particular gene combination responsible for better resistance [77]. Other techniques are related to plant biotechnology and genetic engineering, but these last options are unacceptable in many countries and remain in the laboratory experiments phase [78,79]. As an alternative, increasing attention is being paid to the priming process, i.e., short-lasting pre-exposure of plants to a variety of exogenously applied agents in order to induce a rapid and/or effective defense response to subsequently occurring stress [23,80]. There are many different types of priming methods, which are generally classified into chemical, physical, and biological methods, depending on the source of priming agents. Thus, plants can be primed by natural or synthetic chemical compounds (e.g., phytohormones), by physical factors such as (non-)ionizing radiations, and by colonization with beneficial microorganisms such as bacteria and mycorrhizal fungi [81]. Moreover, priming can be applied to various organs and at various stages of the plant life cycle. The most frequently used is seed priming, which provides faster and more uniform seed germination, ensures efficient nutrient and water uptake, releases photo- and thermo- dormancy, as well as improves seedling vigor in relation to their further growth and yield under both optimal and adverse conditions [82,83,84]. Less often, priming concerns seedlings, young plants, or their parts although they show significantly greater tolerance to different abiotic stresses than untreated ones [85,86]. Importantly, priming acts on the phenotypic level without any permanent DNA modification, and therefore its effects can be reversed [22]. Moreover, its performance can vary in respect to plant species, temperature, priming duration, priming agents, and their concentration [81].

**Figure 2:**
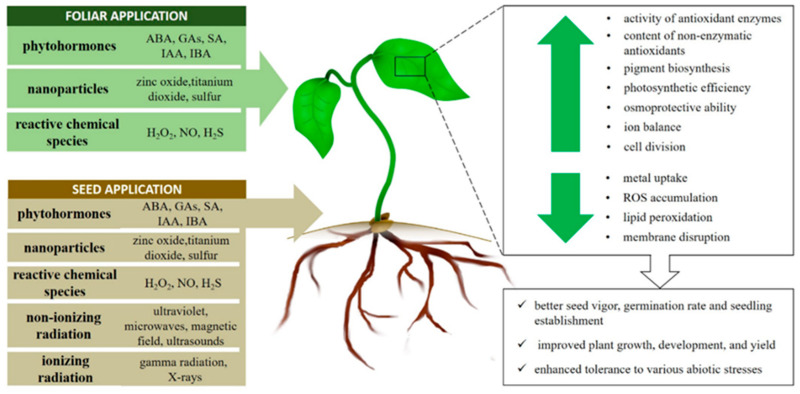
Types of exogenously applied priming agents discussed in this review, as well as their possible applications and the beneficial consequences of treatments that protect plant cells and provide improved defense potential.

Currently, priming seems to be the most promising approach for the mitigation of abiotic stress due to various possibilities regarding application. In the present review, we have briefly summarized the latest achievements in the techniques which have attracted the greatest interest recently. Their types, and the general mode of action discussed in this text, are shown in Figure 2.

### 3.1. Chemical Priming Agents

Chemical priming is one of the most popular strategies and one which has some advantages. One of them is versatility, since chemical compounds work in a broad number of species and improve tolerance to multiple stress types. Furthermore, chemical agents might be applied directly to selected plant tissues/organs, or during specified developmental stages, in order to minimize growth inhibition [87]. It is a good technique, especially for producing tolerant plants when more conventional methods are difficult to perform [88]. On the other hand, little is still known about the impact of priming agents on ecosystems and their persistence in the environment, although it is anticipated that their application may be a widespread tool in agriculture in the near future. The mode of action for three main groups of chemical priming agents, the ones that are most frequently used, is presented below on the basis of the latest scientific achievements.

#### 3.1.1. Phytohormonal Priming

As has been shown in recent years, exogenous application of phytohormones may increase the metabolic status of plants in response to various abiotic and biotic stresses. In this respect, **abscisic acid** (ABA) is one of the more promising priming agents. The effectiveness of ABA lies in both reducing the ROS pool and activating non-enzymatic and enzymatic ROS scavenging. In research conducted by Saha et al. [89], seedlings of two rice genotypes were pre-treated with 10 µM ABA for 24 h and then exposed to arsenite (As (III)). In contrast to the untreated control, seedlings of both ABA-primed genotypes had reduced accumulation of superoxide anion (O_2_^•−^) and hydrogen peroxide (H_2_O_2_) under arsenite toxicity. Furthermore, lipid oxidative damage, measured by 2-tribarbituric acid reactive substances (TBARS), was reduced by 25% and 48% under metal stress for ABA-treated individual genotypes compared to non-pre-treated ones. Mitigation of oxidative stress in primed seedlings was associated with higher concentrations of total glutathione, non-protein thiols, cysteine, and phytochelatins, as well as the increased activity of glutathione reductase [89]. Similarly, previous studies by Rehman et al. [90] and Leng et al. [16] demonstrated that Cd inhibited plant growth parameters; whereas the application of ABA (10 μM ABA) on seedlings considerably counteracted the Cd-caused negative effect and improved the root length, plant height, and biomass of shoots and roots of mung bean. Also in this case, the enhanced growth of Cd-stressed individuals sprayed with ABA was due to modification of the antioxidant defense systems. Interestingly, it is supposed that leaves-applied ABA can be then transferred to roots in order to regulate the response of the whole plant to metal stress [90,91,92]. ABA may also act positively on growth and physiological parameters under alkaline stress via effective control of ROS homeostasis, as found for alfalfa seedlings in which the enhanced activity SOD and POD was observed [93]. Furthermore, a significant increase in Ca^2+^ and Mg^2+^ content, as well as higher Ca^2+^/Na^+^ and Mg^2+^/Na^+^ ratios, was noticed in primed seedlings under alkaline conditions. In addition, genes encoding some important proteins involved in the sequestration of Na^+^ in vacuoles, i.e., vacuolar Na^+^/H^+^ exchanger (NHX) and vacuolar H^+^-PPase (AVP), which might help in neutralization of its excess amount, were overexpressed in primed seedlings [93].

Recent studies showed that, as well as ABA, priming with **gibberellins** (GAs) has a positive effect on plant growth under stress conditions. A study by Ahmad et al. [39] showed that foliar application of GA_3_ (1 µM) on chickpea seedlings resulted in the increased activity of antioxidant enzymes (SOD, CAT, GST), which provided effective ROS scavenging and reduced membrane disruption, thus ensuring tolerance to Cd stress. The better response of plants treated with GA_3_ to Cd presence can be also attributed to the reduced uptake and translocation of toxic ions, as well as increased accumulation of nutrient minerals (Ca, Na, Mg, K, Cu, P, Fe) [39]. This could possibly be achieved through the regulation of H^+^-ATPase activity, as shown for soybean [94]. The advantageous impact of GA_3_ application on morpho-physiological parameters and stress mitigation was also determined in *Lolium perenne* under Ni and Cd exposure and in *Lepidium sativum* under As treatment [95,96].

The latest articles also indicate the beneficial role that priming with **salicylic acid** (SA) has regarding plant tolerance to abiotic stresses; however, in the case of this phytohormone, seed priming seems to be the most effective technique. It has been recently found that the soaking of wheat seeds in SA at a concentration of 100 µM for 24 h results in significant improvements in germination rate and growth parameters in the presence of chromium (Cr) and Zn due to the prevention of ROS imbalance associated with the increase in the concentration of non-enzymatic antioxidants, mainly AsA and GSH [97]. In turn, SA-primed sunflower seeds exposed to Zn showed better germination properties because the exogenous SA application modulated the endogenous profile of the phytohormones [98]. It was noted that concentrations of SA and GA were increased, while ABA accumulation was inhibited as a result of the overexpression of genes related to SA and GA biosynthesis and the decrease in the expression of ABA-related genes that occurred in combination with a simultaneous increase in the expression of genes engaged in the catabolism of this phytohormone. Additionally, the role of SA in metal stress mitigation may also result from the upregulation of genes encoding proteins related to ion transport, such as heavy metal ATPases and metal tolerance protein (MTP), the overexpression of which provided a reduced accumulation of Zn in sunflowers [98] and Cr in tomato [99]. In these latter examples, seed soaking or foliar spraying with SA at a concentration of 0.5 mM ameliorated growth and the physiological reaction to Cr, in respect to chlorophyll biosynthesis and photosynthetic efficiency, through modulation of the ascorbate-glutathione (AsA-GSH) cycle that contributes to a decline in ROS accumulation and lipid peroxidation [99].

It is well known that the exogenous application of auxin, especially **indole-3-acetic acid** (IAA), or its precursors improves growth and development of plants; however, its role in the mitigation of metal stress is not fully understood, and the physiology of these tolerance mechanisms remains largely unknown. Nevertheless, a study by Mir et al. [85] revealed that foliar application of IAA (at a dose of 10 nM) on *Brassica juncea* plants under Cu stress significantly mitigated adverse responses due to the activation of cell division and elongation, as well as lateral root formation in which this phytohormone is involved. Furthermore, in *B. juncea* plants sprayed with IAA, effective ROS scavenging was observed which, together with improved photosynthesis and chlorophyll fluorescence parameters, sugar metabolism, and N, P, and K content, led to biomass accretion [85]. In turn, priming with indole-3-butyric acid (IBA), an IAA precursor, provided antioxidant protection through the stimulation of glutathione peroxidase activity and greater accumulation of nitric oxide (NO) that effectively reduced the elevated level of superoxides and organic peroxides in the root cells of barley seedlings under Cd stress [100].

#### 3.1.2. Nanoparticle Priming

Nanotechnology is an emerging field with potentially wide-ranging applications in agriculture. The use of nanoparticles (NPs) in plant production, as well as in enhancing plant growth under stressful conditions, including those related to environmental pollution with heavy metals, has increased significantly in recent years [101,102]. Several studies on the seed priming of various plant species with **zinc oxide NPs** (ZnO NPs) have been published, with results indicating the beneficial effects of this NP on germination and growth. Wheat seeds primed with ZnO NPs (at a concentration of 10 mg/L) exhibited better germination rates and vigor index values compared to untreated seeds. In seeds primed with ZnO NPs, increased α-amylase activity was observed that could facilitate the efficient mobilization of starch reserves. Moreover, in plants 30 days after seed priming, increased photosynthetic pigment content (chlorophyll *a*, chlorophyll *b*, and total chlorophylls) and improved photosynthetic efficiency compared to untreated plants were determined. Additionally, the use of nanopriming had a positive effect on redox homeostasis in wheat plants [103]. ZnO nanoparticles, sodium selenite (Na-selenite), sodium selenate (Na-selenate), and their combinations as priming agents for direct-seeded rice seeds were also investigated [84]. It was observed that all tested combinations of the priming agents (10 µmol ZnO-NPs; 50 µmol Na-selenite; 50 µmol Na-selenate; and the following combinations at the mentioned concentrations: Na-selenite + Na-selenate; ZnO-NPs + Na-selenite; ZnO-NPs + Na-selenate; ZnO-NPs + Na-selenite + Na-selenate) resulted in the early emergence of seedlings with increased vigor compared to the control. Furthermore, in the field experiment, all tested combinations improved the plant growth parameters and yield, which was the result of increased photosynthetic pigments, increased phenol and protein content, and the increased uptake of nutrients such as N, P, and K [84]. Salam et al. [80] showed that priming maize seeds with ZnO NPs nanoparticles (500 mg/L for 24 h) significantly improved plant growth, biomass, and photosynthesis efficiency under cobalt (Co) stress. In this case, priming also caused a reduction in ROS accumulation and lipid peroxidation due to increased antioxidant activity in maize shoots. Additionally, priming with ZnO NPs reduced the toxic effect of Co by reducing its absorption. More importantly, the ultrastructures of cell organelles, guard cells, and stomatal aperture were stabilized and able to reduce the adverse effects of Co stress. In turn, the study by Zafar et al. [86] showed the effect of seed priming and the foliar application of Zn NPs (0.1–0.3%) on spinach salinity tolerance. It was found that external use of ZnNPs enhanced the growth of spinach plants, as well as improved biochemical parameters under stress conditions compared to untreated plants. Seed soaking and foliar application of ZnNPs provided a decline in H_2_O_2_ content accompanied by the activation of enzymatic and non-enzymatic antioxidant defense systems, as well as simultaneous accumulation of osmolytes.

The positive effect of priming was also demonstrated in the case of **titanium dioxide NPs** (TiO_2_ NPs). Shah et al. [104] investigated the effect of seed priming with TiO_2_ NPs on the germination and growth of maize seedlings under salinity conditions. Priming with TiO_2_ NPs (60 ppm) resulted in improved germination percentage and energy, improved seedling vigor index values, increased root and shoot length, and improved fresh and dry weights of seedlings. Moreover, priming increased the activity of antioxidant enzymes and ROS scavenging capacity. This experiment showed that priming with TiO_2_ NPs reduced the adverse effects of salinity stress in maize seedlings, as evidenced by a reduction in membrane lipid peroxidation and the relative electrolyte leakage level.

Recently, the effect of priming sunflower seeds with **sulfur NPs** (S NPs) on the cellular defense of seedlings against manganese (Mn) toxicity was also investigated. Priming with S NPs (50 and 100 µM) had a significant impact on reducing oxidative damage caused by excess H_2_O_2_, which was reflected in decreased lipid peroxidation. In primed seedlings, the values of these parameters under Mn stress were similar to those observed in seedlings growing under the control conditions [105].

#### 3.1.3. Priming by Reactive Chemical Species

A significant amount of research has confirmed that the pre-treatment of plants or seeds with low concentrations of reactive oxygen, nitrogen, and sulphur species (such as H_2_O_2_, sodium nitroprusside (SNP), one of the donors for NO, or sodium hydrosulfide (NaHS), a donor for hydrogen sulphide (H_2_S)) strengthens their resilience to later stress events [106,107,108,109]. Improved resistance to abiotic stress may be due to the fact that, at low concentration, these compounds can act as a stress signal transduction which induces stress acclimation and alleviates abiotic stress injury [87]. They play a significant protective role, mainly due to the induction of tolerance to oxidative stress caused by drought, salinity, temperature, or metal toxicity [107,110]. On the other hand, too high a concentration of these reactive chemical species results in oxidative burst and damage to cellular compounds [87].

The exogenously sourced H_2_O_2_ (at a concentration ranging from 100 to 500 μM) has the potential to counteract the toxicity of metallic trace elements in a number of plants, and its mode of action was briefly summarized in some review articles, such as those written by Hossain et al. [111] and Cuypers et al. [112] which discussed H_2_O_2_ interaction with signaling components (e.g., transcription factors, phytohormones, mitogen-activated protein kinases) as well as its involvement in the regulation of ROS homeostasis and gene expression during metal stress. Based on various studies, it can be assumed that the positive effects of H_2_O_2_ priming prior to metal exposure include the reduced accumulation of ROS accompanied by an enhanced activity of antioxidant enzymes, such as SOD, CAT, GPX, APX, and GST, as well as elevated levels of reduced forms of non-enzymatic antioxidants such as GSH and AsA [111]. This may be related to proactive protection of the thiol groups present in proteins that are particularly exposed to oxidation under stressful conditions [113]. Besides suppressing oxidative damage, the accumulation of GSH plays a role in metal detoxification in the cytosol through direct ion binding to thiol groups of its cysteine residues and acts as a precursor of metal-chelating phytochelatins [112]. Indeed, the reduced translocation of Cd ions from root to shoot was demonstrated in *Oryza sativa* cultivars pre-treated with H_2_O_2_ [114]; whereas an opposite result was obtained for Cr in *Brassica napus* seedlings in which foliar application of H_2_O_2_ increased metal movement from roots to aerial organs [115].

Interestingly, more and more recent studies concern the simultaneous application of H_2_O_2_ and other compounds in order to explore their cumulative role in metal stress resilience. As an example, the combination of H_2_O_2_ with 24-epibrassnolide (EBL), an effective by-product from brassinolide biosynthesis, provided tolerance and helped *Solanum lycopersicum* plants to cope well with Cu stress [58]. The positive morpho-physiological response of tomato to Cu treatment was related to a decreased accumulation of these metallic ions in the roots and shoots. Such an effect resulted from the complementary action of both applied molecules, since H_2_O_2_ may affect the absorption and transport of excess Cu ions to above-ground organs due to Cu precipitation at the root surface and preferentially affect the uptake of Ca; whereas EBL improves the accumulation of K, Ca, Fe, and Mg, which are translocated to younger leaves to minimize oxidative damage in photosynthetic machinery [58]. Although, in the study conducted by Nazir et al. [58], H_2_O_2_ and EBL were implemented through distinct modes, i.e., root dipping and foliar spraying, respectively, they both minimized ROS content (H_2_O_2_ and O_2_^•−^) and electrolyte leakage in Cu-stressed plants by modulating the activities of antioxidant enzymes (CAT, POD, SOD) and providing osmotic adjustment through increased storage of proline. In turn, Verna and Prasad [116] investigated the involvement of H_2_O_2_ and NO when applied jointly in the regulation of Cd toxicity in cyanobacteria (from genera of Nostoc and Anabena). Their findings demonstrated the synergistic action of both molecules towards the improved growth and enhanced tolerance of cyanobacteria to Cd. In this case, H_2_O_2_ and NO reduced the intracellular content of Cd through an increased secretion of exopolysaccharides, which make a slimy physical barrier against ion penetration into the protoplast. Furthermore, tested cells were characterized by a well-operating antioxidant defense system, and ROS homeostasis was provided by the enhanced activity of antioxidant enzymes and the endogenous content of reactive nitrogen species that indirectly responded to the balancing of antioxidants in order to cope up with Cd stress [116].

Taking into account other abiotic stresses, research by dos Santos Araújo et al. [117] showed that H_2_O_2_ promoted salt tolerance in maize by protecting chloroplast ultrastructures, as reflected in more efficient photosynthetic performance. Furthermore, plants treated with 15 mM H_2_O_2_ and then exposed to salinity showed increased accumulation of metabolites, such as arabitol, glucose, asparagine, and tyrosine, which may contribute to the maintenance of osmotic stability and reductions in oxidative stress [117]. The role of H_2_O_2_ in salt stress prevention can also be attributed to ion homeostasis. After priming, a decline in Na^+^ and Cl^−^ content in the leaves of sunflowers was observed during salinity stress, as well as positive control of K^+^ and NO_3_^−^ uptake [109]. In turn, the beneficial activity of H_2_O_2_ and NO towards drought stress was noticed by Habib et al. [107]. Despite stress conditions, pre-treated wheat plants exhibited increased growth and grain yield as a result of osmolyte storage and the effective functioning of an antioxidant defense mechanism, leading to a reduced accumulation of H_2_O_2_ and membrane lipid peroxidation [107]. Drought stress effects on agronomic features of plants were also minimized in the case of *Oryza sativa* after both seed soaking and foliar spraying with H_2_O_2_ [118]. Regardless of the application form, rice plants pre-treated with this molecule showed improved yield components such as tiller numbers, number of panicles, number of filled grains, filled grain weight, and harvest index [118].

The beneficial role of **exogenously applied NO**, used in the form of donor compounds (mainly SNP) due to its gaseous nature, has also been well-documented. In experiments that involve increasing stress tolerance, NO is applied the most frequently via foliar spraying [119] or seed soaking [120] at a concentration of 50 µM to 200 µM. Similar to other reactive chemical species, NO can prevent the spread of oxidative stress in cells. As an example, SNP enhanced the activity of enzymatic antioxidants and the AsA-GSH cycle in soybean cultivars under Cu stress [121]. The alleviation of Co stress by foliar-applied SNP in *Lactuca sativa* var. *capitata* resulted in a notable reduction in H_2_O_2_ and malonyldi-aldehyde (MDA) content, enhanced accumulation of photosynthetic pigments, and biomass accretion that was accompanied by the better nutritional status of plants [122]. In turn, Basit et al. [120] studied the impact of SNP under Cr stress on rice seedlings. It has been shown that seed priming improved carbon assimilation and minimized oxidative damage, since NO-treated plants were characterized by lower accumulation of oxidative markers (such as H_2_O_2_, O_2_^•−^, and MDA) and electrolyte leakage as compared to control plants. Consequently, their morphological traits were also improved [120]. It was also proven that NO stimulated seed germination and counteracted the inhibitory effect of Cd and Pb (and salinity as well) on the root growth of *Lupinus luteus.* Additionally, in this case, the increased activity of antioxidant enzymes, mainly SOD which is responsible for the neutralization of O_2_^•−^, was correlated with a decreased level of ROS [123]. Although it would appear most likely that NO modulation of antioxidant enzyme activities and phenol and flavonoid production provides stress amelioration, Hassanein et al. [124] observed the opposite tendency in *Lupinus albus* subsp. *termis* in response to SNP and Ni treatment. Therefore, it was postulated that NO may act as an antioxidant molecule, interacting directly with ROS and giving rise to a number of reactive nitrogen species and their derivatives, which are rapidly degraded to nitrite and nitrate [124]. This is in accordance with the study by He et al. [125] which found that, regardless of aluminum presence, SNP significantly suppressed the generation of O_2_^•−^ and H_2_O_2_ by mitochondria in peanut root tips.

The addition of NO (pre-sowing and foliar) can also minimize the adversaries of salinity stress, not through the activation of antioxidant machinery, but mainly due to osmotic adjustment and Na ion homeostasis. As an example, NO-increased tolerance in broccoli was associated with higher amounts of proline and glycine betaine keeping water potential in cells below the external solution under stress conditions [57]; whereas, in wheat, the antagonistic uptake of toxic Na^+^ with key mineral elements, such as N, K, and Ca, reduced the deleterious effects of salt [119]. Importantly, Alnusairi et al. [119] showed that the application of NO may dismiss salt stress-mediated ravaging by the overexpression of genes encoding both antiporters that are responsible for excluding Na ions from the cytosol to outside the plasma membrane or inside the vacuole (SOS1/NHX1), and aquaporin (AQP) as well as osmotin (OSM-34) which are involved in the maintenance of proper plant–water relations. In turn, the protective effect of exogenous NO under drought stress may be dose-dependent. Majeed et al. [108] found that a foliar spray of 100 µM of SNP markedly improved water status and chlorophyll content and alleviated drought-induced oxidative damages through increased antioxidant enzyme activities (CAT, APX, SOD) in maize hybrids. Moreover, an exogenous supply of SNP increased nitrite and nitrate reductase activities and upregulated GR, GST, and GPX compared to plants not supplied with SNP [108]. In contrast, higher SNP doses (150 and 200 µM) intensified the toxic effects of oxidative stress through increased MDA, H_2_O_2_, and NO content and inhibited the enzymatic activities of antioxidants.

Many studies have indicated the significant role of **H_2_S** priming in the response of plants to various abiotic factors [126]. It has been reported that pre-treating seedlings or a mature plant with NaHS as a H_2_S donor may increase the tolerance of the plant upon following exposure to heavy metals such as Pb, Ni, and As [96,127]. Although many studies have assessed the positive effect of the pre-treatment of seedlings or mature plants with H_2_S in relation to enhancing plant tolerance, few studies have employed H_2_S for seed priming. Valivand et al. [128] reported that seed priming with Ca^2+^ and NaHS influenced the induction of cross-adaptation in seedlings under Ni stress. The authors reported that seed priming with H_2_S and Ca^2+^ triggered signaling pathways, which resulted in the systemic accumulation of dormant stress memory in embryo cells in seeds. Upon subsequent exposure to Ni ions, stress memory was activated and primed plants showed enhanced tolerance-related responses, e.g., enhanced AsA-GSH cycle activity, redox homeostasis, and expression of phytochelatin genes [128]. In turn, Zanganeh et al. [127] reported that pre-treatment with NaHS, applied separately and together with SA, reduced Pb toxicity and improved Fe homeostasis in maize plants. The mechanism of their action was related to modulation of the glyoxalase system consisting of enzymes detoxifying methylglyoxal, which is a potent reactive cytotoxin capable of a complete disturbance of cellular roles, including oxidation of lipids and proteins [127,129].

Christou et al. [130] studied the effect of NaHS (100 μM for 48 h) on the tolerance of strawberry plants to subsequent exposure to salinity. Pre-treatment of roots resulted in increased leaf chlorophyll fluorescence, stomatal conductance, and leaf relative water content, as well as lower lipid peroxidation levels. Additionally, synthesis of NO and H_2_O_2_ in leaves was reduced and high ascorbate and glutathione redox states were maintained. The observed positive changes correlated with the stimulated gene expression of antioxidant enzymes (cytosolic APX, CAT, MnSOD, GR), enzymes involved in ascorbate and glutathione biosynthesis (glutamylcysteine synthetase; L-galactose dehydrogenase; glutathione synthetase), a transcription factor (DREB), and salt overly sensitive (SOS) pathways (SOS2-like, SOS3-like, SOS4) [130]. Hydrogen sulfide pre-treatment (500 μM NaHS for 72 h) also mitigated growth inhibition and regulated root architecture under salt stress in *Malus hupehensis* seedlings, not only through the activation of antioxidant defense (mainly CAT and POD activities), but also through maintaining the balance of water (by proline accumulation) and Na^+^/K^+^ (by higher uptake of K than Na ions) as well [131]. Under drought conditions, H_2_S may improve tolerance by regulating stomatal closure and reducing water loss thanks to ABA synthesis and signaling, which was noticed in *Oryza sativa* seedlings together with an increase in endogenous H_2_S production and antioxidant capacity [132]. However, an innovative approach in the use of H_2_S as a priming agent is its application in combination with NO. In this respect, NOSH is a novel hybrid synthetic compound that simultaneously releases NO and H_2_S. Antoniou et al. [133] demonstrated that NOSH synthetic compounds provide significant protection in *Medicago sativa* plants against drought stress. This protection appears to be achieved through a coordinated modification of improved physiological performance, reactive oxygen/nitrogen species homeostasis, and transcriptional regulation of defense-related pathways [133].

### 3.2. Physical Priming

Priming with physical factors includes a number of methods, especially those related to radiation. Among them, both non-ionizing radiations, such as UV radiation, microwaves, magnetic field radiation, and sonication, and ionizing radiations, i.e., X-ray radiation and γ-radiation, can be distinguished [134]. Physical priming is considered to be an accessible, affordable, and eco-friendly technique which brings beneficial effects on seed parameters, the metabolic activities of plants, and plant development and growth [135]. It has the advantage, over chemical priming, that it does not pollute the environment, which is an important aspect in agriculture, especially if the contemporary injudicious application of chemical compounds during food production is taken into account [136]. Therefore, until recently, physical treatment was successfully employed in crops, mainly for stimulating seed germination and seedling establishment since these stages are considered to be the most critical stages in the life cycle and ultimately determine field production. This aspect of physical priming application has been widely discussed over the past few years [6,134,135,136,137].

Despite increasing understanding of the effects of physical priming performance on plants under optimal conditions, data in the literature on its application to alleviating stress nuisance are still limited, especially in respect to metal toxicity. Thus, in the present review we have focused on the latest achievements that are related mostly to drought and salinity, during which physical priming strengthens antioxidant response. It is therefore likely that physical treatment of plants subsequently exposed to excess amounts of metallic trace elements will bring comparable responses. Nevertheless, the mode of the physical agent’s actions in plants under metal stress is also mentioned whenever the most recent studies were available.

#### 3.2.1. Priming with Non-Ionizing Radiation

**Ultraviolet (UV)** radiation is a type of electromagnetic radiation with a vibration frequency between 30 PHz and 750 THz, photon energy between 3 and 124 eV, and a wavelength between 10 and 400 nm, which is shorter than visible light but longer than X-ray radiation. UV radiation is divided into UV-A, UV-B, and UV-C, with UV-A radiation being the least harmful to living organisms and UV-C the most [134]. Both seed and seedling UV-B priming, applied for 45 min at 4 kJ/m^2^ intensity, was shown to effectively alleviate oxidative stress and its resulting damage by significant reductions in superoxide, H_2_O_2_, and MDA content in stress-sensitive rice variety (*Oryza sativa* cv. Aiswarya) seedlings under stressful conditions caused by NaCl, PEG, and UV-B treatments [138]. The study also demonstrated that UV-B priming led to significant increases in glutathione and ascorbate contents, SOD, CAT, and APX activity, gene expression levels, photosystem activities, foliar gas exchange parameters, and, finally, in mitochondrial activity. The increases were the most pronounced in seedlings subjected to NaCl stress. Similar results were reported for UV-B primed seeds for two varieties of rice: Neeraja and Vaisakh. Additionally, reductions in leaf osmolarity level, increases in proline, total sugar, and free amino acids content, and induced expression levels of stress-related proteins (Hsp90 and Group 3 late embryogenesis abundant proteins) under NaCl and PEG stress were observed [139]. The observed differences were significantly higher in the tolerant variety (Kanchana) than in the sensitive one (Aiswarya) and were also reported by Thomas et al. [138], who found that UV-B priming at low doses (4 and 6 kJ/m^2^) led to increased levels of flavonoids and anthocyanins, the increased activity of phenylalanine ammonia lyase, and increased levels of cuticular wax in rice seedlings under UV-B, NaCl, and PEG stress. The UV-C seedling priming of a cumulative dose of 10.2 kJ/m^2^ was also found to significantly reduce leaf spot disease severity in strawberry plants due to induced accumulation of pathogenesis-related proteins, terpenes, phenolic compounds with triggered ROS, and antioxidant enzymes, while also inducing plant hormone synthesis [140]. Moreover, the transgenerational effect of UV-B priming was shown by the rice seedlings of the drought-tolerant Vaisakh variety being characterized by the increased expression of genes encoding antioxidant enzymes and stress-related proteins in F0 generation, with even more of an increase in the F1 generation after re-priming. This resulted in better protection against PEG stress [141]. The UV-B priming protection against UV-B stress was proven to be related to the UV RESISTANCE LOCUS (UVR8) pathway in *Arabidopsis thaliana*, since 14-day old seedlings without UVR8, primed for 10 min with UV-B at 35 µW/cm^2^, did not acquire UV-B resistance [142].

**Microwave radiation** is a form of electromagnetic radiation with a frequency ranging between 300 MHz and 300 GHz [143]. Physical seed priming with microwave radiation at 2.45 GHz for a short time had stimulatory effects on seed germination, seedling growth, and biomass accumulation in different cereals, such as barley, rice, and wheat [144]. A study by Bian et al. [145] proved that treatment of *Fagopyrum tataricum* with microwaves with a power of 300 W and a frequency of 2.45 GHz for 75 s optimally increased the activity of antioxidant enzymes (SOD, CAT, POD, and APX), leading to the increase in the total reduction potential of plants and the ability of the seedlings to neutralize radicals such as 2,2-diphenyl-1-picrylhydrazyl (DPPH), 2,2’-azino-bis(3-ethylbenzothiazoline-6-sulfonic acid (ABTS), O_2_^•−^, and ^•^OH [145]. Microwaves have been used as factors for improving the resistance of crops to a number of stress factors. Maswada et al. [146] applied microwave priming prior to sowing two *Triticum aestivum* genotypes, Giza 168 and Gharbiya. The results of the conducted experiments proved that microwave priming (with 700 W of power, a variable frequency of 2.45 GHz, and a wavelength of 125 nm with a power intensity of 126 mW/cm^2^) improved wheat resistance to drought, increasing both the yield and growth parameters through improvement of tissue water content and a reduction in membrane permeability. Furthermore, osmotic adjustment and decreased H_2_O_2_ accumulation through increasing proline content and ROS scavenging activity were also observed [146]. In turn, Farid et al. [147] showed that microwaves can also help to alleviate heavy metal stress in *Brassica napus*. Pre-saw treatment of the genotype Faisal Canola (RBN-03060), with microwaves with a frequency of 2.45 GHz for 30 s, led to a greater ability to grow and biomass accretion for plants treated with Ni. Heavy metal-stressed plants also showed a higher concentration of photosynthetic pigments, including chlorophyll *a* and *b* and carotenoids, and higher antioxidant enzyme activity (SOD, POD, APX, CAT), which was associated with a reduction in ROS (H_2_O_2_) content and the oxidative damage caused by them (MDA, electrolyte leakage). Moreover, it was shown that microwave priming resulted in greater accumulation of Ni from the soil, especially in roots, stems, and leaves [147].

**Magnetic fields** can be used for priming in several variants: as alternative magnetic field (AMF), electromagnetic field (EMF), pulsed magnetic field (PMF), static magnetic field (SMF), and sinusoidal magnetic field (SSMF) priming. All of those techniques were used in research on crops, and their application improved the germination and vigor of plants, as well as the response to unfavorable environmental factors, although SMF is the most common one [148]. Mohammadi and Roshandel [149] applied SMFs of 90 mT, 200 mT, and 250mT on *Hyssopus officinalis* plants for 5 min. The best effect was obtained at 200 mT. In response to drought stress, the plants subjected to magnetopriming showed higher dry matter content, total chlorophyll and phenol content, and a higher reduction capacity (DPPH, O_2_^•−^ scavenging) resulting from, among other factors, higher CAT, APX, and GPX activity. At the same time, magnetopriming led to a reduction in oxidative damage to biological membranes, which reduced electrolyte leakage [149]. Kataria et al. [150] applied an SMF of 200mT on soybean for 1 h, which resulted in increased resistance to salinity. Plants subjected to magnetopriming showed greater leaf area, leaf mass, photosynthetic activity, and nitrogenase activity than plants subjected to salinity stress only. However, the content of H_2_O_2_ and AsA, and the activity of antioxidant enzymes, was reduced due to magnetopriming. These changes resulted in higher biomass accumulation, yield, and harvest index values for soybean under both the saline and non-saline conditions [150]. Baghel et al. [151] showed that the use of a 200 mT SMF for 1 h on *Zea mays* plants reduced their susceptibility to salinity. Magnetopriming increased the content of photosynthetic pigments, as well as increasing photosynthesis parameters such as the quantum yield of PSII photochemistry (F_v_/F_m_), electron transport per leaf CS (ETo/CSm), the density of reaction centers (RC/CSm), and the performance index (PI). Moreover, the maize leaves showed lower H_2_O_2_ accumulation, which proves the reduction in oxidative stress. These changes resulted in better plant growth and increased maize yield under salinity conditions [151].

**Ultrasound priming** involves treating plants or seeds with the energy of acoustic waves with a frequency greater than 20 kHz [134]. Xia et al. [137] applied high-intensity ultrasound (HIU) with a frequency of 28 kHz and a power of 17.83 W/cm^2^ for 5 to 30 min on brown rice seeds. Ultrasound priming led to both an increase in starch content and a simultaneous reduction in the size of grains and in the content of reducing sugars. Moreover, the accumulation of free amino acids, γ-aminobutyric acid, antioxidants, and proline (as stress-responsive secondary metabolites) may also have potentially positive effects on plant response to adverse environmental factors [137]. Dashab and Omidi [152] primed *Brassica napus* with ultrasound at 40 kHz and 59 kHz with a power of 60, 80, and 100 W for 2, 4, 6, 8, and 20 min. Depending on the combination of those parameters, different physiological effects were achieved. The greatest increase in seed germination was observed with 40 and 59 kHz at 100 W for 2 min of exposure, while an increase in vigor and seedling weight was observed with 59 kHz at 100 W. At 40 kHz with 80 W and an exposure time of 8 min, an increase in the content of photosynthetic pigments was determined [152]. In turn, Rao et al. [135] treated canola cultivars Youyanzao18 and Zaoshu104 for 1 min with ultrasound at a frequency of 20 kHz in order to reduce susceptibility to Cd stress. It has been shown that ultrasound, depending on the Cd dose, can improve such parameters as germination, shoot and root length, and fresh mass. Moreover, in the Youyanzao18 cultivar, ultrasound priming increased the activity of SOD, POD, CAT, and APX, as well as the increased content of proline, GSH, and soluble protein. This translated to a reduction in MDA content, which indicates less oxidative damage to biological membranes in response to Cd. In both cultivars, ultrasound increased pods per plant, seeds per pod, and rapeseed yield. Importantly, the accumulation of Cd in all parts of the plant decreased [135]. Similarly, Chen et al. [153] demonstrated that ultrasonic vibration can help wheat seedlings eliminate an excess amount of ROS resulting from Cd and Pb treatment, as well as improve the biosynthesis of molecules and division of cells, leading to biomass accretion despite the metal stress.

#### 3.2.2. Priming with Ionizing Radiation

**Gamma (γ) radiation** is a high-energy type of ionizing radiation capable of penetrating and interacting with living tissues, whose absorbed dose is expressed in units of Gray (Gy). Usually, Cobalt-60 is used for this type of priming [144]. When Hussein [83] used 5, 10, and 20 Gy gamma radiation on barley plants, it was shown that both lower doses improved plant growth and yield, while the highest one (20 Gy) increased shoot growth and tiller number; however, only at the lowest radiation dose (5 Gy) was an increase in the content of photosynthetic pigments observed. Gamma radiation enhanced the accumulation of phenols, flavonoids, free amino acids, and antioxidant enzymes (APX, POD, CAT), but it also elevated H_2_O_2_ content. Moreover, it led to a reduction in the content of sugars and proline [83]. Researchers have proven that low doses of γ-rays not only modify redox homeostasis, but they also change the protein pattern and the metabolic profile in plants, leading to improved growth and yielding. As an example, a study by Hanafy and Akladious [154] showed that a dose of 100 Gy improved growth and yield for *Trigonella foenum-graecum* plants, as well as the content of soluble proteins in leaves and the content of phenols and flavonoids. Moreover, a significant rise in the content of AsA, α-tocopherol, retinol, and proline was observed. In turn, the highest dose of radiation (400 Gy) caused a decrease in the content of all tested parameters and induced changes in the DNA profile that consisted of the appearance and disappearance of polymorphic bands [154]. Pradhan et al. [155] used gamma radiation at a dose of 10 Gy on the microalgae *Chlamydomonas reinhardtii* (which is considered to be a model organism for studying the effects of heavy metals on photosynthetic organisms) and exposed it to Cd stress. As a result of Cd treatment, redox homeostasis was disturbed due to a decline in antioxidant enzyme activity and in the content of photosynthetic pigments. Consequently, cell death was induced and growth was minimized. On the contrary, the application of γ-radiation had positive effects on the mentioned parameters, and cell growth and biochemical synthesis were not injured. As a consequence, an increased resistance to toxic Cd ions was achieved [155].

**X-rays** are characterized by a wavelength ranging from 0.01 to 10 nm of the electromagnetic spectrum, which corresponds to frequencies ranging from 30 to 30,000 PHz and energies oscillating from 120 eV to 120 keV [144]. Currently, there are very few new studies concerning the effects of this type of priming on plants; however, in 2019, Rezk et al. [82] used X-rays in doses from 0 to 100 Gy on two genotypes of okra (*Hibiscus esculentus*), genotypes of Hassawi and Clemson. It was shown that radiation doses up to 5 Gy improved plant morphological parameters, the content of photosynthetic pigments, the activity of antioxidant enzymes (CAT, SOD, APX), and the content of low-molecular weight antioxidants (AsA, GSH, anthocyanins). In contrast, higher doses of radiation (at levels above 5 Gy) had the opposite effect, and plants treated with this type of priming showed greater lipid peroxidation caused by the increased concentration of ROS (mainly H_2_O_2_ and O_2_^•−^) [82]. This confirmed the previous discoveries of Al-Enezi et al. [156] regarding the influence of X-rays on date palm (*Phoenix dactylifera* cv. Khalas). In this case, the inhibitory impact of radiation on seed germination was noticed even at a dose of 0.25 Gy, and a graduated increase in X-ray dose up to 15 Gy contributed to further reductions in germination; however, at the same time, an increase in root length was observed. A similar stimulatory effect was found for the leaf length of the date palm plants, but it concerned only X-ray doses between 0.05 and 0.25 Gy [156]. It can be summarized that only low doses of this type of radiation may improve plant growth parameters, but little is still known about its ameliorative actions under various stress conditions and further research is therefore required.

## 4. Concluding Remarks

In the present review, we have briefly discussed the adaptative traits of metallophytes, whose application may be an antidote to environmental pollution with heavy metals, perceived as one of the most dangerous factors for all living organisms. The amazing biology of metallophytes, especially in respect to metal detoxification and accumulation, as well as tolerance to drought and salinity, make them applicable for the phytoremediation and reclamation of chemically degraded areas which, after returning to their original state before contamination, can be reused for different goals. Furthermore, deeper insight into plants with evolutionarily developed tolerance mechanisms, may help to obtain specimens with ideal survival levels and fertility under stressful conditions. It seems to be particularly important to take into account that the majority of plants do not exhibit tolerance to abiotic stresses developing as a result of severe selection pressure due to the complexity associated with the inheritance of adaptive traits. Therefore, to combat the most important global problems, including metal pollution, drought, and salinity, through biological methods and to provide sustainable agriculture and food security for continuing global population growth, increasing attention is being given to priming strategies which make plants capable of responding more effectively and more rapidly to stress. Since priming offers a large variety of priming factors, doses, and application forms, the diversified morphological and biochemical responses of plants can be observed. Thus, the chemical and physical treatment for stress amelioration requires extensive future research for the elaboration of specific protocols in respect to optimal dosage and duration of exposure, which certainly vary between genotypes and environmental conditions. Furthermore, further understanding of both the mode of actions of particular priming agents and the mechanisms underlying the better performance of primed plants can lead to combined usage of various priming methods, preferably with synergistic effects that would allow a reduction in the dose of each agent compared to the dose used individually. Undoubtedly, the joint knowledge gathered here clearly indicates that all priming agents contribute to the scavenging of excess amounts of ROS via efficiently operating antioxidant machinery and thus put oxidative mitigation at the core of enhanced tolerance to various stressors.

## Figures and Tables

**Table 1:** Examples of the usefulness of halophytes in particular phytoremediation techniques for the removal of various metallic trace elements.

Technique	Halophyte Species	Accumulated Metal (s)	References
phytostabilization	*Atriplex atacamensis*	As	[62]
	*Atriplex halimus*	Cd, Pb	[67]
	Cochearia species	Zn, Pb	[63]
	*Halimione portulacoides*	Zn, Cu, Ni, Co	[73]
	*Tamarix hispida*	Zn, Pb	[64]
phytoextraction	*Chenopodium botrys*	Cd	[68]
	* Halogeton glomeratus *	Cr, Ni, Cu, Zn, As, Cd, Hg	[74]
	*Limoniastrum monopetalum*	Cd, Pb	[66]
	*Sesuvium portulacastrum*	Cr, Cd, Cu, Zn	[75]
	*Tamarix gallica*	As	[76]

## Data Availability

Not applicable.
